# High-grain feeding causes strong shifts in ruminal epithelial bacterial community and expression of Toll-like receptor genes in goats

**DOI:** 10.3389/fmicb.2015.00167

**Published:** 2015-03-02

**Authors:** Jun-hua Liu, Gao-rui Bian, Wei-yun Zhu, Sheng-yong Mao

**Affiliations:** Department of Animal Nutrition and Feed Science, Laboratory of Gastrointestinal Microbiology, College of Animal Science and Technology, Nanjing Agricultural UniversityNanjing, China

**Keywords:** high-grain feeding, bacterial community, ruminal epithelium, Toll-like receptors, goat

## Abstract

High-grain (HG) feeding used in intensive goat production can affect the physiology of the rumen wall, but the changes induced in the epimural bacterial community and host Toll-like receptors (TLRs) are not well understood. In this study, 10 male goats were randomly allocated to two groups and fed either a hay diet (0% grain; *n* = 5) or an HG diet (65% grain; *n* = 5). The changes in the ruminal epithelial bacterial community and expression of TLRs during long-term (7 weeks) HG feeding were determined using pyrosequencing and quantitative real-time polymerase chain reaction. Principal coordinate analysis and analysis of molecular variance (AMOVA) results showed that HG feeding caused a strong shift in bacterial composition and structure. At the genus level, our data revealed that it increased the relative abundance of taxa *Butyrivibrio*, unclassified *Clostridiales*, *Mogibacterium*, unclassified *Anaerolineaceae*, and *Succiniclasticum*, and decreased the proportion of unclassified *Ruminococcaceae*, unclassified *Rikenellaceae*, unclassified *Erysipelotrichaceae*, *Howardella*, and unclassified *Neisseriaceae*. The HG-fed goats also exhibited upregulation of the relative mRNA expression of TLR2, TLR3, and TLR5 in the rumen epithelium (*P* < 0.05). Correlation analysis revealed that the increase in TLR expression was associated with changes in the relative abundance of ruminal epithelial bacteria. This study provides a first insight into the adaptive response of ruminal epithelial bacterial populations to HG feeding in goats and shows that these changes were associated with alterations in TLR expression. These findings provide new insight into understanding of host–microbial relationships in ruminants.

## Introduction

In current intensive goat production, to meet the energy demand for fast-growing goats, high-grain (HG) diet feeding has become common practice in the nutritional management of meat goats in China. It is well known that HG diet feeding affects ruminal fermentation characteristics and the structure of the content-associated rumen microbial population (Russell and Rychlik, 2001; Callaway et al., 2010; Hook et al., 2011; Metzler-Zebeli et al., 2013; Petri et al., 2013; Zened et al., 2013). However, similar information regarding ruminal epithelial (epimural) bacteria is incomplete compared to knowledge of the bacterial community in rumen content.

Through the use of electron microscopy and culture-dependent (McCowan et al., 1978, 1980; Cheng and Wallace, 1979) and culture-independent (Cheng et al., 1980; Sadet et al., 2007; Li et al., 2012) techniques, it has been found that epimural bacteria are different from those associated with rumen contents in dairy cattle, steer, and sheep. The epimural bacterial community has some specific functions, such as hydrolysis of urea, scavenging of oxygen, and recycling of epithelial tissue (Cheng and Wallace, 1979; Wallace et al., 1979; Dinsdale et al., 1980; Petri et al., 2013). Some studies have demonstrated, using 16S rRNA sequencing, polymerase chain reaction and denaturing gradient gel electrophoresis (PCR-DGGE), and quantitative reverse transcription PCR (qRT-PCR) analysis (Sadet-Bourgeteau et al., 2010; Chen et al., 2011), that ruminal epithelial bacterial population and diversity can be affected by dietary changes. Nevertheless, information on epimural bacterial community numbers has been limited, due to the low throughput of the traditional 16S rRNA clone library method and fingerprint profiles. Determining 16S rRNA short variable tags using a high-throughput sequencing technology such as 454 pyrosequencing has provided an unprecedented sequencing depth, with tens to thousands of reads per sample. As a result, there has been renewed interest in measuring and comparing the composition and richness of microbial taxa in epimural samples in ruminant. However, only one published report to date has investigated changes in epimural bacterial richness and diversity in HG-fed heifers using high-throughput pyrosequencing techniques (Petri et al., 2013). In addition, little is known about the changes in epimural bacterial community of meat goats during HG diet feeding, especially depending on the high-throughput technique.

Toll-like receptors (TLRs) make up a group of pattern recognition receptors that are commonly identified in the gastrointestinal tissues of monogastric animals (Abreu, 2010; Malmuthuge et al., 2012). Recently, Malmuthuge et al. (2012) reported that TLRs1–10 can express in the rumen epithelium of calves. It is well known that TLRs can recognize the sensing of host epimural bacteria and bacterial products and trigger an immune response, which is critical for maintaining host–microbial homeostasis in monogastric animals (Hooper et al., 2012). Dysregulation of this process can cause chronic inflammatory and epithelial barrier dysfunction (Abreu, 2010; Ulluwishewa et al., 2011). Our previous study demonstrated that HG diet feeding caused massive barrier dysfunction and local inflammation in the rumen epithelium (Liu et al., 2013). If results from monogastric intestinal tissues are applied to the rumen epithelium, it seems that HG-induced rumen epithelial barrier dysfunction is associated with dysregulation of host TLR signaling. Furthermore, the changes in rumen epithelial TLR expression might be linked to the host epimural bacterial community (Chen et al., 2012). Thus, in the present study, we hypothesized that (1) HG feeding can cause an alteration in epimural bacterial community and (2) such a change might be associated with variation in the rumen epithelial TLRs expression. Therefore, the objective of this study was to investigate changes in the richness and diversity of epimural bacterial communities and the expression of host TLR genes during HG feeding in goats.

## Materials and methods

The experimental design and procedures were approved by the Animal Care and Use Committee of Nanjing Agricultural University, in compliance with the Regulations for the Administration of Affairs Concerning Experimental Animals (The State Science and Technology Commission of P. R. China, 1988).

### Animals, diets, and experimental design

This animal experiment was carried out at the experimental station of Nanjing Agricultural University in Jiangsu Province, China. The experimental design was previously described in detail (Liu et al., 2013). Briefly, ten rumen-cannulated, castrated male goats (Boer × Yangtze River Delta White) aged 2–3 years were used in this experiment. Prior to the experiment, all of the goats were fed pure hay *ad libitum* for 5 weeks to ensure adaptation to the low-energy hay diet. The goats were then randomly assigned to groups fed either a hay diet (Hay; *n* = 5) or an HG diet (HG; *n* = 5), and placed in individual pens (1.2 × 1.2 m) with free access to water. Adaptation to the HG diet was carried out over 5 d with progressively increasing amounts of grain for 65%. The diet (750 g dry matter per animal per day) was offered in equal amounts at 08:30 and 16:30 daily for 7 weeks. Hay fed in this experiment originated from one batch. The metabolic energy citation(ME) intake of the goats in the hay group (30 kg body weight; 8.31 MJ/kg dry matter) was slightly above the requirement for maintenance, and that of the goats in the HG group (30 kg body weight; 11.31 MJ/kg dry matter) permitted a growth rate of 200 g/day according to guidelines on the nutrient requirements of goats (NY/Y816-2004; Ministry of Agriculture of China, 2004). The nutrient compositions of the hay and HG diets are summarized in Supplementary Table 1. No significant differences in body weight gain (0.30 ± 0.26 vs. 1.02 ± 0.52 kg, *P* = 0.248) or dry matter intake were observed between the hay and HG groups during the feeding trial.

### Sample collection

On day 50, 4–5 h after the last feeding according to the normal continuous feeding protocol, the goats were euthanized by captive bolt stunning followed by exsanguination from the carotid arteries. Within 5 min, a segment of the rumen epithelial tissue was collected and immediately washed three times in ice-cold phosphate-buffered saline. The samples were divided into two portions. The first portion of the tissue sample was cut into smaller pieces (approximately 0.5 × 0.5 cm) and immediately frozen in liquid nitrogen for RNA extraction. The second tissue sample portion was cut to approximately 2 × 2 cm and scraped from the underlying tissue using a germ-free glass slide, immediately transferred into liquid N, and then stored at −80°C until DNA extraction. Washed rumen papillae were immediately fixed in 2.5% glutaraldehyde for microscopic analysis.

### Morphometric analysis

Fixed rumen papillae was prepared for electron microscopy using methodology reported by Graham and Simmons (2005). Samples were then examined using scanning electron microscopy (Hitachi Model S-3000N, Hitachi Technologies, Tokyo, Japan) and transmission electron microscopy (Hitachi H-7650, Hitachi Technologies, Tokyo, Japan).

### Real-time quantitative PCR (qPCR)

Total RNA was extracted from the rumen epithelium tissue with acid using TRIzol (Takara Bio, Otsu, Japan), as described by Chomczynski and Sacchi (1987). The RNA concentration was then quantified using a NanoDrop spectrophotometer (ND-1000UV-Vis; Thermo Fisher Scientific, Waltham, MA). The absorption ratio (260/280 nm) of all of the samples was between 1.8 and 2.0, indicating high RNA purity. Aliquots of RNA samples were subjected to electrophoresis through a 1.4% agarose–formaldehyde gel to verify integrity. The concentration of RNA was adjusted to 1 μg/μl based on optical density and stored at −80°C. Total RNA (1 μg) was reverse-transcribed using a PrimeScript® RT Reagent Kit with gDNA Eraser (Takara Bio) according to the manufacturer's instructions.

The primers used for TLR2 (forward – 5′-CTGTGTGCGTCTTCCTCAGA-3′ and reverse – 5′-TCAGGGAGCAGAGTAACCAGA-3′), TLR3 (forward – 5′-TCTTTTCGGGACTGTTGACC-3′ and reverse – 5′-AAATCCCCCATCCAAGGTAG-3′), TLR4 (forward – 5′-GGTTTCCACAAAAGCCGTAA-3′ and reverse – 5′-AGGACGATGAAGATGATGCC-3′), and TLR5 (forward – 5′-TCAATGGGAGCCAGATTTTC-3′ and reverse – 5′-CCTTCAGCTCCTGGAGTGTC-3′) were described by Charavaryamath et al. (2011), and the primer used for GAPDH (forward – 5′-GGGTCATCATCTCTGCACCT-3′ and reverse – 5′-GGTCATAAGTCCCTCCACGA-3′) was described by Wang et al. (2009). All of the primers were synthesized by Invitrogen Life Technologies (Shanghai, China). Real-time qPCR of the target genes and GAPDH was performed using an ABI 7300 real-time PCR system (Applied Biosystems, Foster City, CA) with fluorescence detection of SYBR green dye. Amplification conditions were as follows: 95°C for 30 s followed by 40 cycles of 5 s at 95°C and 31 s at 57.5°C (for GAPDH) or 62°C (for the TLRs). Each sample contained 1–10 ng cDNA in 2 × SYBRGreen PCR Master Mix (Takara Bio) and 200 nmol/L of each primer in a final volume of 20 μL. All measurements were performed in triplicate. A reverse-transcription-negative blank of each sample and a no-template blank served as negative controls. The relative amount of each studied mRNA was normalized to GAPDH mRNA levels as a housekeeping gene, and the data were analyzed according to the 2^−ΔΔCT^ method.

### Microbial DNA isolation

One gram of underlying rumen epithelial tissue was used for DNA extraction. The DNA was extracted by a bead-beating method using a mini-bead beater (Biospec Products, Bartlesville, OK), followed by phenol–chloroform extraction (Sun et al., 2008). The solution was precipitated with ethanol, and the pellets were suspended in 50 μL Tris-EDTA buffer. The DNA samples were quantified using a NanoDrop spectrophotometer (Nyxor Biotech, Paris, France).

### DNA pyrosequencing

Bacterial amplicons were sequenced using the 454 GS FLX Titanium chemistry at the Majorbio Bio-Pharm Technology Co., Ltd., Shanghai, China. PCR amplification of the V1–V3 region of bacterial 16S rRNA was performed using universal primers (27F 5′-AGAGTTTGATCCTGGCTCAG-3′, 533R 5′-TTACCGCGGCTGCTGGCAC-3′) incorporating FLX Titanium adapters and a sample barcode sequence. The primers were synthesized by Invitrogen Life Technologies (Shanghai, China). The PCR amplification was conducted in a 50 μL C1000 thermal cycler (Bio-Rad, USA) containing 10 μL 5-fold reaction buffer, 50 ng of total genomic DNA, 0.4 μM of each primer, 0.5U Pfu polymerase (TransStart-FastPfu DNA Polymerase, TransGen Biotech, Beijing, China) and 2.5 mMdNTPs. The cycling parameters were as follows: 5 min initial denaturation at 95°C; 25 cycles of denaturation at 95°C (30 s), annealing at 55°C (30 s), elongation at 72°C (30 s); and final extension at 72°C for 5 min. Three separate PCR reactions of each sample were pooled for pyrosequencing. Amplicons from the two microbial groups were quantified fluorometrically, normalized per sample and pooled per microbial group. A total of 1 μg DNA of each of the four resulting pools was loaded onto agarose gel (1% wt: vol). Bands were visualized and excised under blue light transillumination, and amplicons were gel purified using a QIAquick Gel Extraction Kit (Qiagen, Hilden, Germany). Amplicons were then quantified using a Quant-iT PicoGreen dsDNA Assay Kit (Invitrogen, Carlsbad, CA) according to the manufacturer's instructions. Equal concentrations of amplicons were pooled from each sample. Emulsion PCR of pooled samples was done with the GS FLX Titanium Lib-L LV emPCR Kit (Roche Applied Science, Indiananpolis, IN) following the manufacturer's recommendations.

### Pyrosequencing data analysis

The sequences were processed using the MOTHUR program (Schloss et al., 2009, 2011; version 1.29.0; University of Michigan; http://www.mothur.org/wiki/). 16S rRNA reads were decoded based on the 5 bp sample-specific barcodes and processed to remove poor-quality sequences. To reduce sequencing errors, the shhh.flows command was applied, which is the MOTHUR implementation of the AmpliconNoise algorithm (Quince et al., 2011). Quality filters were applied to trim and remove sequences with the following characteristics: less than 200 bp in length; average quality score less than 35; homopolymers longer than eight nucleotides; and more than two different bases to the primer. In order to obtain a non-redundant set of sequences, unique sequences were determined, and used to align against the SILVA reference alignment database (Pruesse et al., 2007); chimeras were removed using chimera.uchime (http://drive5.com/uchime); sequences identified as being of eukaryotic origin were removed; the candidate sequences were screened and pre-clustered to eliminate outliers; and a distance matrix was generated from the resulting sequences. Sequences were clustered into operational taxonomic units (OTUs) using the furthest neighbor algorithm. Representative sequences from OTUs at a 0.03 distance were obtained and classified using the Ribosomal Database Project's Bayesian classifier (Wang et al., 2007). Rarefaction curve was generated at the level of 3%, which was calculated by the distance-based OTU (Schloss et al., 2011), and Rarefaction curves and Good's coverage were calculated to quantify the coverage and sampling effort. Community diversity was estimated with the unnormalized reads using the based coverage estimator (ACE), Chao1, and Shannon indices. The unweighted UniFrac distance method (Lozupone and Knight, 2005) was used to perform a principal coordinates analysis (PCOA) with all OTUs, and a distance-based analysis of molecular variance (AMOVA) was conducted to assess significant differences between samples.

The 16S sequencing data for all the samples analyzed in this study was submitted to the Sequence Read Archive (SRA; http://www.ncbi.nlm.nih.gov/Traces/sra/), under accession SRP052769.

### Statistical analysis

Statistical analysis were carried out by conducting tests using the SPSS software package (SPSS version 16, SPSS, Inc.). The normality of the distribution of variables was tested using the Shapiro–Wilk test. The microbial diversity data, some data of taxa richness, and the relative mRNA expression of TLRs and cytokines found to have a normal distribution were analyzed by the independent-samples *t*-test procedure, according to the following model: Y = μ + C + e, where μ is the mean, C is the effect of diet, and e is the residual error. The Kruskal–Wallis test was used to analyze variables found to have a non-normal distribution (some data of taxa richness), and the statistical model used: $H=\frac{12}{n(n+1)}\sum_{i\;=\;1}^{k}\frac{Ri^{2}}{n_{i}}-3(n+1)$ where H is the Kruskal-Wallis test; *n* is the number of measurements; *R_i_* is the sum of the ranks; *n_i_* is the number of experiments. Significance was declared at *P* < 0.05.

Double dendrograms were constructed using the comparative functions and multivariate hierarchical clustering methods of NCSS 2007 (NCSS), on the basis of the abundance of bacterial groups at different taxonomic levels. Clustering with all OTUs was performed using the Ward's minimum variance method with no scaling. Correlations between the relative mRNA expression of TLRs and the relative abundance of epimural bacteria and cytokine mRNA expression were assessed by Pearson's correlation test $\left(\gamma =\frac{\sum XY-\frac{\left(\sum X)-(\sum Y\right)}{n}}{\sqrt{\left(\sum X^{2}-\frac{{\left(\sum X\right)}^{2}}{n})(\sum Y^{2}-\frac{{\left(\sum Y\right)}^{2}}{n}\right)}}\right)$, where γ is the Pearson correlation coefficient; X is the relative mRNA expression of TLRs; Y is the epimural bacteria data found to have a normal distribution or cytokine mRNA expression; *n* is the number of experiments) or Spearman's rank test $(\gamma =1-\frac{6\sum d^{2}}{n^{3}-n}$, where γ is Spearman's Rank correlation coefficient; *d* is the difference in rank between the relative mRNA expression of TLRs and the epimural bacteria data found to have a non-normal distribution, *n* is the number of measurements) depending on data normality using GraphPad Prism version 5.00 (GraphPad Software, San Diego, CA). Significance was declared at *P* < 0.05.

## Results

The body weight gain (0.30 ± 0.26 vs. 1.02 ± 0.52 kg, *P* = 0.248) or dry matter intake were not affected by experimental diet during the feeding trial.

### Ruminal pH and concentrations of volatile fatty acids, lactate, and lipopolysaccharides

The data of rumen fermentation of experimental goats were reported in our previous study (Liu et al., 2013) (Supplementary Table 2). Briefly, HG feeding decreased (*P* < 0.001) the ruminal pH, and increased (*P* < 0.001 to <0.019) the concentrations of propionate, butyrate, valerate, isovalerate, total volatile fatty acids, lactate, and lipopolysaccharides significantly.

### Epimural bacterial community of goats

In total, 55,633 reads were obtained for the 16S rRNA genes in rumen epithelium of all goats, and 38013 reads were valid correspondingly, accounting for 68.3% of their raw reads. The sequences were further analyzed by MOTHUR software, and these valid sequences were classified into 7673 OUTs at sequence divergences of 0.03. The average number of OTUs was 1364 ± 138, with an average coverage of 85.25 ± 1.72%. The Chao1 richness, ACE, and Shannon diversity indexes were on 2953 ± 360, 4764 ± 660, and 6.02 ± 0.13, respectively. Within the bacterial population, 18 phyla were found across all samples. *Firmicutes*, *Bacteroidetes*, and *Proteobacteria*, were the dominant phyla, representing 53.12%, 20.88%, and 13.35%, respectively. *Actinobacteria*, *Chloroflexi*, and *Tenericutes* represented average percentages of 2.79%, 2.25%, and 1.32%, respectively, of the total sequences. The proportion of some phyla (*Spirochaetes*, *Fibrobacteres*, *Synergistetes*, and *Planctomycetes*) was less than 1% of total microbial community, and other phyla (*Lentisphaerae*, *Elusimicrobia*, *Chlamydiae*, *Cyanobacteria*, *Deinococcus-Thermus*, *Fusobacteria*, and *Nitrospirae*) were not consistently present in all of the different host populations. In the *Firmicutes* phylum, the most dominant class was *Clostridia* (average of 48.45%, respectively, of the total sequences), represented mainly by families *Lachnospiraceae*, unclassified *Clostridiales*, and *Ruminococcaceae* (average of 27.14%, 10.48%, and 8.09%, respectively, of the total sequences). In the *Bacteroidetes* phylum, the most dominant class was *Bacteroidia* (average of 20.38%, respectively, of the total sequences), represented mainly by families *Prevotellaceae*, *Rikenellaceae*, and unclassified *Bacteroidales* (average of 10.92%, 4.69%, and 4.51%, respectively, of the total sequences). In the *Proteobacteria* phylum, the major classes were *Deltaproteobacteria* and *Betaproteobacteria* (average of 5.74% and 5.35%, respectively, of the total sequences), represented mainly by families *Desulfobulbaceae*, *Comamonadaceae*, and *Neisseriaceae* (average of 5.51%, 2.90%, and 2.36%, respectively, of the total sequences). At the genus level, 100 taxa were detected over the entirety of the samples; the most dominant genus was *Butyrivibrio* (average of 11.01% of the total sequences), followed by the genera *Desulfobulbus* (5.51%), *Mogibacterium* (4.33%), and *Prevotella* (4.01%). For clarity and visualization purposes, the top 30 bacterial taxa are presented in a heat map (Supplementary Figure 1).

### Effect of HG diet on epimural bacterial diversity and relative abundance levels

**Figure 1:**
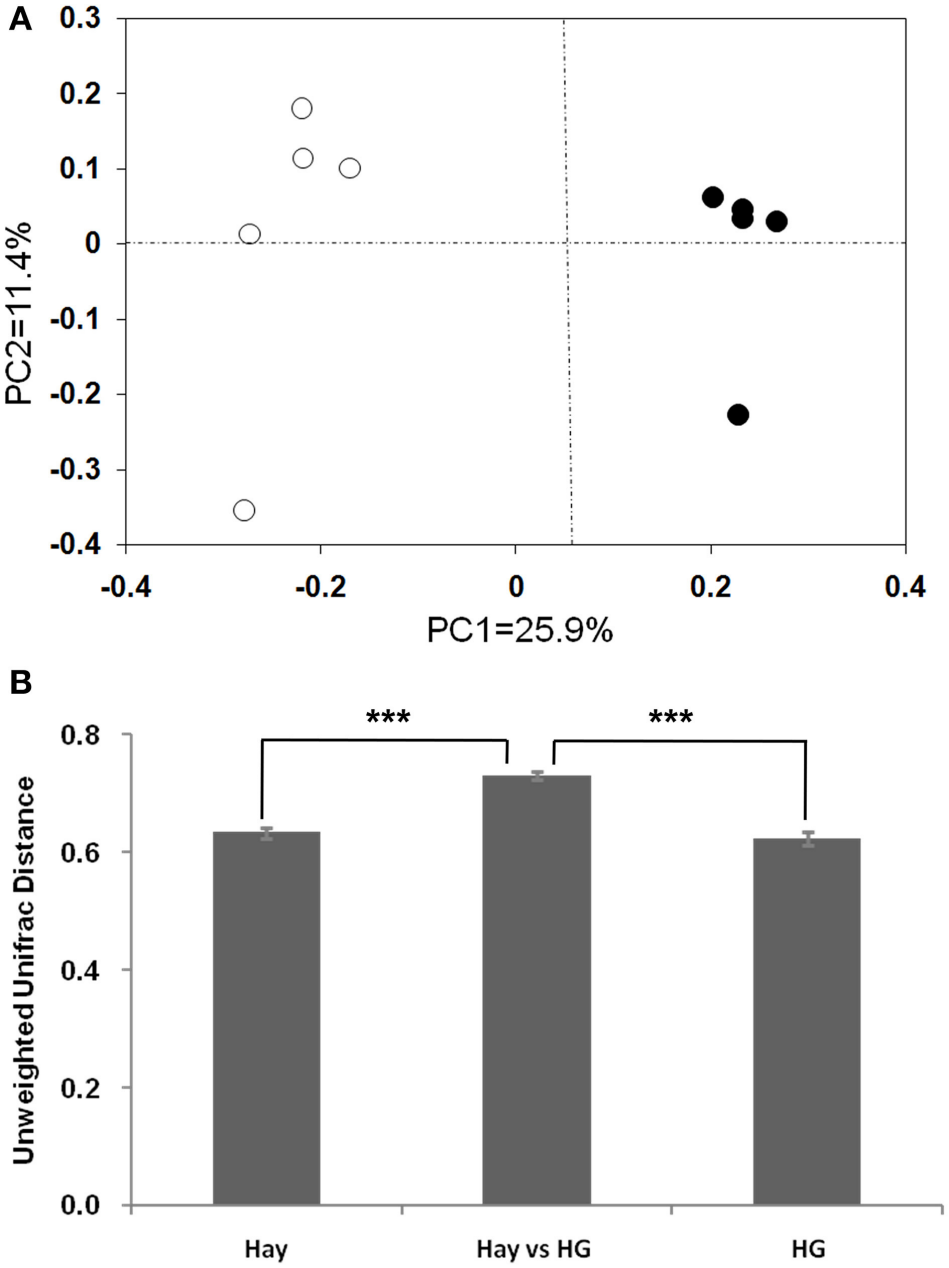
**Differences in the ruminal epithelial (epimural) bacterial structure between hay and high-grain (HG)-fed goats**. **(A)** Unweighted UniFrac principal coordinate analysis (PCoA) of epimural microbiota based on the operational taxonomic unit data from 454 pyrosequencing run. The marks relate to the diets of donor goats: hay diet (°), HG diet (•). **(B)** Mean pairwise unweighted UniFrac distance for subsets of samples (means ± SEM, *n* = 5). Hay, sample-to-sample variation among replicates in hay group; HG, sample-to-sample variation among replicates in HG group; Hay vs. HG, hay vs. HG comparison variation. ^***^P < 0.001.

The rarefaction curves of epimural bacterial communities are shown in Supplementary Figure 2. At dissimilarity levels of 0.03, the rarefaction analysis revealed that HG feeding decreased bacterial diversity in the rumen epithelium compared with the hay group. The unweighted UniFrac metric in MOTHUR was used to evaluate β-diversity across the samples (Figure 1). The PCOA result exhibited that the goats fed the hay diet were distinctly separated from those fed the HG diet in the plot (Figure 1A; axis 1 + axis 2 = 37.3%). The further analysis indicated that the diet significantly affected the epimural microbial communities (AMOVA, Fs = 2.75, *P* < 0.001). Mean pairwise unweighted UniFrac distance showed that there was no significant difference in sample-to-sample variation among replicates between the hay and HG groups (Figure 1B; 0.63 ± 0.01 vs. 0.62 ± 0.01; *P* = 0.482). The hay vs. HG comparison variation was greater than the sample-to-sample variation among replicates in the hay group (Figure 1B; 0.73 ± 0.01 vs. 0.63 ± 0.01; *P* < 0.001) and the HG group (Figure 1B; 0.73 ± 0.01 vs. 0.62 ± 0.01; *P* = 0.482). In addition, the Venn profile revealed that there were 299 unique OTUs belonging to the hay-fed goats and 227 unique OTUs belonging to the HG-fed goats (Supplementary Figure 3).

As is shown in Table 1, the results indicated a decrease in OTU numbers (*P* = 0.016), ACE (*P* = 0.047), and Chao1 richness (*P* = 0.028), but not in community evenness [Shannon's (*P* = 0.117) and Simpson's (*P* = 0.465)] during HG diet feeding. At the phylum level, HG feeding caused a higher proportion of *Firmicutes* (*P* = 0.008) and *Chloroflexi* (*P* = 0.008) and a negative effect on *Actinobacteria* (*P* = 0.008) and *Tenericutes* (*P* = 0.008) phyla; the *Bacteroidetes* and *Proteobacteria* phyla were not affected significantly (*P* > 0.05) (Supplementary Figure 4). At the genus level, a total of 100 taxa were examined, 24 of which exhibited significant variability across diets. There was a significant increase in relative abundance of dominant genera *Butyrivibrio* (*P* = 0.008), unclassified *Clostridiales* (*P* = 0.008), *Mogibacterium* (*P* = 0.008), unclassified *Anaerolineaceae* (*P* = 0.008), *Succiniclasticum* (*P* = 0.008), and *Ruminococcus* (*P* = 0.008) associated with HG feeding, and the proportion of *Butyrivibrio* was almost three times higher in the HG group than in the hay group (Table 2). Conversely, there was a corresponding decrease in the proportion of unclassified *Ruminococcaceae* (*P* = 0.008), unclassified *Rikenellaceae* (*P* = 0.008), unclassified *Erysipelotrichaceae* (*P* = 0.008)*, Howardella* (*P* = 0.008), and unclassified *Neisseriaceae* (*P* = 0.008) (Table 2). All of the analysis results showed that HG feeding had a considerable effect on epimural bacterial composition and diversity.

**Table 1:** **Effects of high-grain (HG) diet feeding on the diversity of ruminal epithelial bacterial community at the 3% dissimilarity level**.

	**OTU**	**ACE**	**Chao 1 value**	**Shannon index**	**Evenness**
Hay	1615 ± 227	5821 ± 1069	3561 ± 599	6.24 ± 0.23	0.77 ± 0.01
HG	1113 ± 56	3708 ± 507	2345 ± 200	5.79 ± 0.05	0.75 ± 0.00
*P*-value	0.016	0.047	0.028	0.117	0.602

*Values shown are means ± SEM, n = 5*.

*OTU, operational taxonomic units; ACE, abundance-based coverage estimator*.

**Table 2:** **Effects of high-grain (HG) feeding on average relative abundance of genus level (% of total sequences) in rumen epithelium, ranked by alphabetical order of first letter of phylum, family and genus name**.

**Phylum**	**Family**	**Genus**	**Abundance (%)**		***P*-value**
			**Hay**	**HG**	
*Actinobacteria*	*Coriobacteriaceae*	Unclassified *Coriobacteriaceae*	3.10 ± 0.54	0.86 ± 0.22	0.008
*Bacteroidetes*	*Prevotellaceae*	*Prevotella*	4.36 ± 0.79	3.66 ± 0.50	0.548
		Unclassified *Prevotellaceae*	5.34 ± 0.39	8.30 ± 0.80	0.056
	*Rikenellaceae*	Unclassified *Rikenellaceae*	5.80 ± 0.56	3.59 ± 0.50	0.016
	Unclassified *Bacteroidales*	Unclassified *Bacteroidales*	5.00 ± 0.25	4.02 ± 0.44	0.095
*Chloroflexi*	*Anaerolineaceae*	Unclassified *Anaerolineaceae*	1.05 ± 0.06	2.84 ± 0.53	0.008
*Firmicutes*	*Lachnospiraceae*	*Acetitomaculum*	2.23 ± 0.34	1.33 ± 0.29	0.095
		*Butyrivibrio*	4.48 ± 0.43	17.55 ± 2.05	0.008
		*Howardella*	3.68 ± 0.53	1.75 ± 0.23	0.008
		*Syntrophococcus*	1.71 ± 0.34	1.13 ± 0.08	0.151
		Unclassified *Lachnospiraceae*	9.82 ± 0.77	9.50 ± 0.78	1.000
	*Ruminococcaceae*	*Ruminococcus*	0.45 ± 0.05	2.20 ± 0.52	0.008
		Unclassified *Ruminococcaceae*	8.36 ± 0.85	4.97 ± 0.77	0.016
	Unclassified *Clostridiales*	*Mogibacterium*	3.30 ± 0.37	5.36 ± 0.56	0.016
		Unclassified *Clostridiales*	4.75 ± 0.72	7.91 ± 0.85	0.032
	*Veillonellaceae*	*Succiniclasticum*	1.16 ± 0.22	3.21 ± 0.35	0.008
	*Erysipelotrichaceae*	Unclassified *Erysipelotrichaceae*	5.08 ± 0.46	2.76 ± 0.31	0.008
*Proteobacteria*	*Comamonadaceae*	*Comamonas*	2.59 ± 0.37	2.69 ± 0.34	1.000
	*Neisseriaceae*	Unclassified *Neisseriaceae*	4.57 ± 2.10	0.14 ± 0.05	0.008
	*Desulfobulbaceae*	*Desulfobulbus*	5.74 ± 1.26	5.27 ± 0.63	1.000
	*Campylobacteraceae*	*Campylobacter*	2.27 ± 0.23	1.96 ± 0.48	0.841
*Tenericutes*	Unclassified *Mollicutes*	Unclassified *Mollicutes*	2.27 ± 0.21	0.30 ± 0.07	0.008

*Only results obtained for the predominant bacterial genera (relative abundance >1% in at least one sample) are presented. Values shown are means ± SEM, n = 5*.

### Core epimural bacterial community

The OTU detected across all samples was defined as the core epimural bacterial community. The results indicated that 16.9% of the OTUs appeared in all samples belonging to *Butyrivibrio* (6.47% of total sequences), *Desulfobulbus* (3.84% of total sequences), unclassified *Erysipelotrichaceae* (2.91% of total sequences), unclassified *Prevotellaceae* (2.90% of total sequences), *Comamonas* (2.45% of total sequences), and *Howardella* (2.26% of total sequences). We also compared the unique OTUs associated with hay and HG diet. In the hay group, 47.2% of the OTUs were found to be unique to this diet. Unclassified *Neisseriaceae* was found to be (4.48% of total sequences) unique in the goats fed the hay diet, but it was not found in HG-fed goats. During the HG diet feeding, 35.9% of the OTUs were found to be unique to this diet: unclassified *Lachnospiraceae* (4.56% of total sequences), unclassified *Clostridiales* (4.19% of total sequences), and *Butyrivibrio* (3.97% of total sequences). Genus *Ruminococcus* (1.32% of total sequences) was found to be unique to the HG-fed goats.

### Effects of HG feeding on relative mRNA expression of TLR and cytokine genes

**Figure 2:**
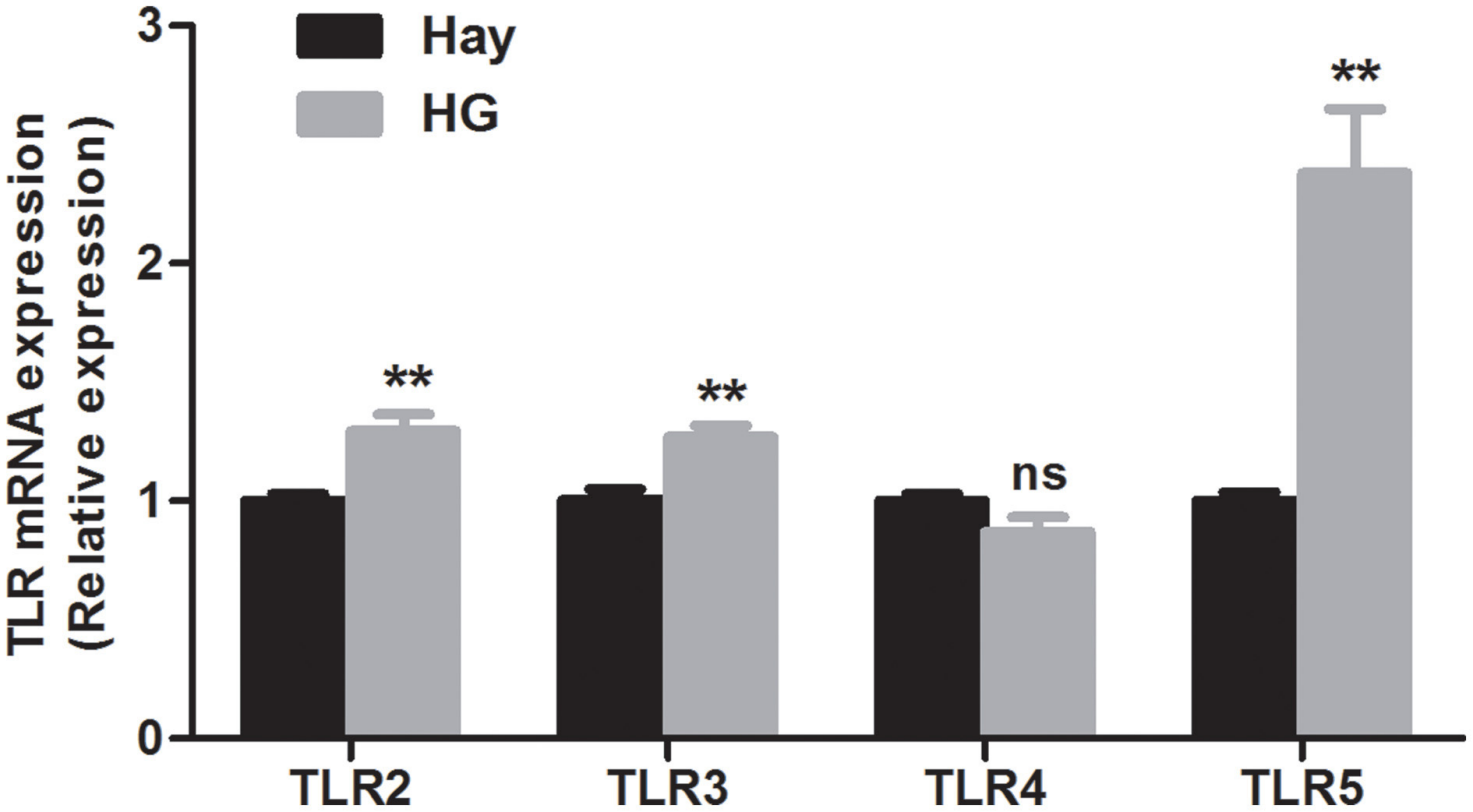
**Effects of high-grain feeding on the relative mRNA expression of Toll-like receptors in the rumen epithelium of goats (means ± SEM, *n* = 5)**. All analyses were performed in triplicate. ^**^P < 0.01, NS: not significant.

The relative mRNA expression of TLR genes, measured by qRT-PCR, are shown in Figure 2. The results revealed that the HG-fed goats indicated upregulation of the relative mRNA expression of TLR2 (*P* = 0.005), TLR3 (*P* = 0.004), and TLR5 (*P* = 0.001) in rumen epithelia compared with the hay-fed goats. HG feeding had no significant effect on the relative mRNA expression of TLR4 (*P* = 0.089). The relative expression of cytokine in rumen epithelium were reported in our previous study (Liu et al., 2013). Briefly, the data exhibited that the relative expression of TNF-α (*P* = 0.017) and IFN-γ (*P* = 0.012) of HG-fed goats were higher than that of hay-fed goats significantly, and HG feeding had no significant effect on the relative expression of IL-1β (*P* = 0.066), IL-6 (*P* = 0.082), and IL-10 (*P* = 0.894) genes.

### Correlation analysis

**Figure 3:**
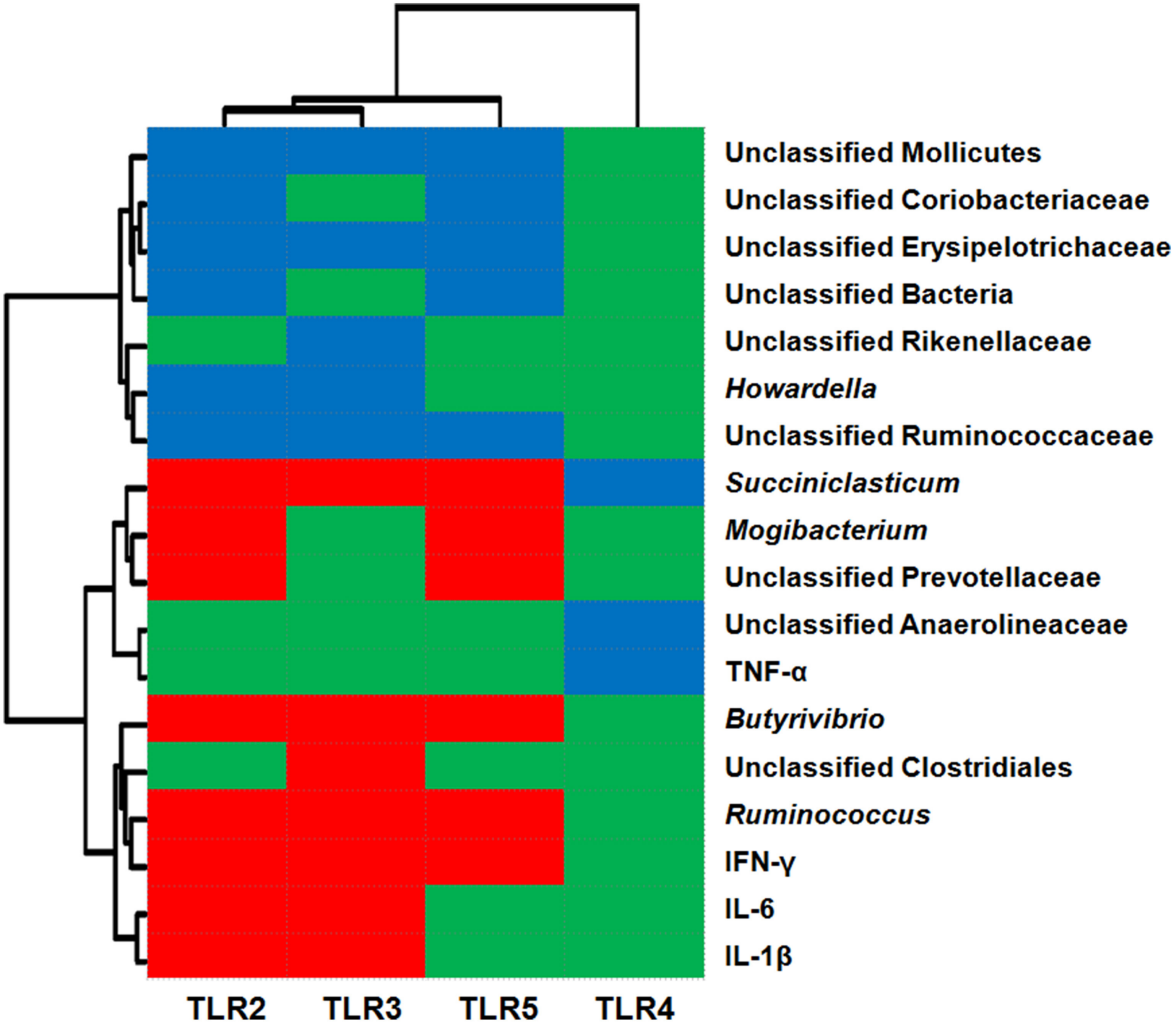
**Correlation analysis between the relative mRNA expression of Toll-like receptors (TLRs) and relative abundance of epithelium-associated microbiota (at the genus level) and mRNA expression of cytokines**. Only results obtained for the predominant bacterial genera (relative abundance >1% in at least one sample) for which the abundance was significantly associated with TLRs are shown. Cells are colored based on Pearson's correlation coefficient. Red represents a significant positive correlation (*P* < 0.05), blue represents a significant negative correlation (*P* < 0.05), and green represents a non-significant correlation (*P* > 0.05).

The relationships between TLR expression and the relative abundance of epimural bacteria and cytokine expression are shown in Figure 3. The data revealed that the abundance levels of 11 taxa were correlated (*P* < 0.05) with the relative mRNA expression levels of TLR2 [six negative (unclassified *Mollicutes*, unclassified *Coriobacteriaceae*, unclassified *Erysipelotrichaceae*, unclassified Bacteria, *Howardella*, and unclassified *Ruminococcaceae*) and five positive (*Ruminococcus*, *Butyrivibrio*, *Mogibacterium*, unclassified *Prevotellaceae*, and *Succiniclasticum*)]; the abundance levels of 10 taxa were correlated (*P* < 0.05) with the relative mRNA expression levels of TLR5 [five negative (unclassified *Mollicutes*, unclassified *Coriobacteriaceae*, unclassified *Erysipelotrichaceae*, unclassified Bacteria, and unclassified *Ruminococcaceae*) and five positive (*Ruminococcus*, *Butyrivibrio*, *Mogibacterium*, unclassified *Prevotellaceae*, and *Succiniclasticum*)]; the abundance levels of eight taxa were correlated (*P* < 0.05) with the relative mRNA expression levels of TLR3 [five negative (unclassified *Mollicutes*, unclassified *Erysipelotrichaceae*, unclassified *Rikenellaceae*, *Howardella*, and unclassified *Ruminococcaceae*) and three positive (unclassified *Clostridiales*, *Ruminococcus*, and *Butyrivibrio*)]; the abundance of one taxa (unclassified *Anaerolineaceae*) was negatively associated (*P* < 0.05) with the relative mRNA expression of TLR4. In addition, the relative mRNA levels of cytokines IL-1β, IL-6, and IFN-γ were positively associated with the relative mRNA expression levels of TLR2 and TLR3 (*P* < 0.05); the relative mRNA expression of cytokine IFN-γ was positively correlated with TLR5 expression (*P* < 0.05); and TNF-α mRNA expression was negatively associated with TLR4 mRNA expression (*P* < 0.05).

## Discussion

At the phylum level, *Firmicutes* (53.12%) were the most abundant bacteria detected in the epimural bacterial community in the goats examined in this study, followed by *Bacteroidetes* (20.88%) and *Proteobacteria* (13.35%). This finding confirmed the findings reported in previous studies that phylum *Firmicutes* are relatively common in tissue-adherent populations (Sadet-Bourgeteau et al., 2010; Chen et al., 2011; Petri et al., 2013). In addition, the relative abundance of phylum *Proteobacteria* was higher than that in rumen content (the relative abundance of phylum *Proteobacteria* was 0.54% in rumen content; unpublished data), which was consistent with previous studies on sheep and heifers (Chen et al., 2011; Petri et al., 2013). The greater abundance of phylum *Proteobacteria* in the rumen epithelium was believed to be caused by the trace amounts of oxygen diffused through the tissue, which might favor a higher density of *Proteobacteria*, as many members of this phylum are microaerophiles or facultative anaerobes, and hence, not sensitive to oxygen toxicity (Sadet-Bourgeteau et al., 2010).

It is well known that diet influences ruminal fermentation characteristics and the structure of content-associated rumen microbial population (Russell and Rychlik, 2001; Sadet et al., 2007; Hook et al., 2011; Huo et al., 2014). However, the effects of diet on the epimural bacterial community remain controversial. A previous study, using PCR-DGGE technology, reported that the epimural community in lambs was less influenced by diet than were the microbiota associated with rumen contents (Sadet et al., 2007). Using PCR-DGGE and qRT- PCR analysis, Chen et al. (2011) demonstrated that an HG diet affects the diversity, but not density, of epimural bacteria in heifers. Petri et al. (2013) demonstrated, through pyrosequencing, that less than 5% of the total OTUs identified exhibited significant variability across diets in heifers. In the present study, the data revealed that HG feeding decreased epimural bacterial diversity and caused a strong shift in bacterial structure and composition. Furthermore, the Venn profile indicated that there were 299 unique OTUs belonging to the hay-fed goats and 227 unique OTUs belonging to the HG-fed goats. These results, which provide evidence that HG feeding influences the epimural bacterial community, are somewhat inconsistent with those of previous studies (Chen et al., 2011; Petri et al., 2013).

There are several possible reasons for the discrepancies between our results and those of previous studies. First, the differences in ruminant species might contribute to the variations in the epimural bacterial changes detected. Because the epimural bacteria inhabit the host tissues, it is suggested that the host species might play a role in regulating bacterial diversity and density. In the previous studies, the strong host effect might have masked the diet effect in sheep and heifers (Sadet et al., 2007; Sadet-Bourgeteau et al., 2010; Chen et al., 2011). The individual difference of goats was smaller than that of sheep and cattle (Liu et al., 2013), and thus, the diet effect masked by the host effect might be smaller in the present study. Second, variations in the type of grain in the diet might also have resulted in the differences in epimural bacterial response. Wheat was grain component for the sheep study (Sadet-Bourgeteau et al., 2010) and barley used as the concentrated diet in the heifer studies (Chen et al., 2011; Petri et al., 2013), while corn and wheat were the grain components in our present study. The differences in ruminal degradation of these cereal grains might contribute to the variations in epimural bacterial response. Third, and perhaps most important, we must take into consideration the HG-induced changes in rumen epithelial structure when comparing our results to those of Petri et al. (2013). Our previous study demonstrated that the HG diet used in this study caused parakeratosis, budding, and extensive sloughing of the stratum corneum (Liu et al., 2013), which potentially increases the availability of attachment sites for opportunistic bacteria (Plaizier et al., 2012) (Supplementary Figure 5). In addition, many previous studies have demonstrated that parakeratosis is linked with quantitative changes in the epimural microbial flora (Semjen et al., 1982; Steele et al., 2009). Thus, disruption of the rumen epithelial structure might account for the increased effect in the present study. Finally, the differences in analysis techniques, DNA extraction methods and sampling methods between our study and previous studies may also play a role in the discrepancies.

Most of the effects of the HG diet were observed at the genus level. The present study found that an HG feeding supports a higher proportion of members of several genera in the rumen epithelium, including *Butyrivibrio*, *Succiniclasticum*, *Mogibacterium*, and *Ruminococcus* (Table 2). The effect of the HG diet on the abundance of *Succiniclasticum*, *Mogibacterium*, and *Ruminococcus* was consistent with the results of Petri et al. (2013). However, the effect of HG feeding on the abundance of *Butyrivibrio* was inconsistent with the results of that study, in which the dietary change from a low-grain to an HG diet were much larger than ours (Petri et al., 2013). Those researchers found that HG feeding decreased the relative abundance of *Butyrivibrio fibrisolvens* in the rumen epithelium. This discrepancy might be associated with the differences in diet formulation and ruminant species, as mentioned earlier. Also, previous study demonstrated many *Butyrivibrio* species are amylolytic (Cotta, 1988), which may be an important potential reason for the increase in the abundance of rumen epithelial *Butyrivibri*o during HG feeding in the present study. Furthermore, as epithelial *Butyrivibrio* release butyrate close to the epithelium, they might enhance butyrate bioavailability for the host, which is useful for rumen epithelial proliferation (Sakata and Tamate, 1978; Shen et al., 2004). Therefore, an increase in the relative abundance of *Butyrivibrio* in the rumen epithelium might be an adaptation response to repair a damaged rumen epithelium by increasing epithelial proliferation during long-term HG feeding. *Mogibacterium* are anaerobic, Gram-positive, non-fermentative bacteria, first identified in the rumen epithelium by Li et al. (2012); the function of this genus in the rumen epithelium is unknown. Many previous studies have reported that this genus was commonly associated with periodontal disease and infected root canals in the human mouth (Nakazawa et al., 2000; Sato et al., 2012). Furthermore, Chen et al. (2012) reported that *Mogibacterium* was enriched in the colonic mucosa of colorectal cancer patients. Thus, these findings imply that the increase in the abundance of *Mogibacterium* during HG feeding could have some deleterious effects on rumen epithelial health. *Ruminococcus* are Gram-positive bacteria that were once considered to be cellulose-degrading bacteria in rumen content. Some *Ruminococcus* species are able to degrade mucin and are linked to gastrointestinal disease in humans (Crost et al., 2013; Wang et al., 2013). However, the physiological roles of *Ruminococcus* associated with rumen epithelium are unknown. This indicates the need for more research on the interactions between rumen epimural bacterial community and rumen epithelium function.

It is well known that TLRs can recognize the sensing of host epimural bacteria and bacterial products and trigger immune responses critical for maintaining host–microbial homeostasis in monogastric animals (Hooper et al., 2012). Dysregulation of this process can result in chronic inflammation of the gastrointestinal tract (Abreu, 2010). In addition to reporting changes in the bacterial composition of the rumen epithelium, this study, for the first time, report changes in the mRNA expression of TLRs in the rumen epithelium of goats during HG feeding.

Although the function of these epimural bacteria is not yet fully understood (Chen et al., 2012), the observed correlations between relative abundance of epimural bacteria and expression of the TLR genes support our speculation that epimural bacteria play a role in stimulating the innate immune response of the rumen epithelium in goats. Our study demonstrated that the HG diet increased the relative expression levels of TLR2, TLR3, and TLR5. A previous study indicated that TLR-2 is mainly involved in responses to cell wall components of Gram-positive bacteria (Melmed et al., 2003). Therefore, the increased expression of TLR2 might be dependent on the overgrowth of Gram-positive bacteria *Butyrivibrio*, *Succiniclasticum*, *Mogibacterium*, and *Ruminococcus* members of the Firmicutes. TLR-5 has a role in the recognition of bacterial flagellin (Rhee et al., 2005; Abreu, 2010). Thus, the observed upregulation of TLR5 expression might be dependent on the overgrowth of bacteria rich in flagellin, such as *Butyrivibrio*. Furthermore, many studies on humans have indicated that TLR5 is expressed only on the basolateral surface, where it can trigger the production of cytokines in response to basolateral flagellin (Abreu, 2010). Another study demonstrated that luminal flagellin was only able to activate TLR5 after injury to the epithelial barrier (Rhee et al., 2005). Thus, the epithelial barrier also determines whether the bacteria and their products access the basolateral surface and trigger TLR5 expression. In our previous study, the HG diet increased rumen epithelial permeability (Liu et al., 2013), allowing bacteria or bacterial products from the rumen lumen to enter the mucosal tissue, which can stimulate host immune responses. Our correlation results revealed that the relative mRNA expression of TLR5 was positively associated with IFN-γ production. Therefore, the observed differences in TLR5 expression and antimicrobial defense molecules between the two diets might be a result of increased permeability that allows the exposure of pattern recognition receptors and secretory molecules to epimural bacteria. It has also been reported that altered epithelial permeability is associated with changes in gastrointestinal microbes, which can subsequently affect host metabolism and rumen health (Ulluwishewa et al., 2011).

It has been reported that epimural bacterial composition regulates the expression of epithelial TLRs (Hooper et al., 2012), which triggers the production of cytokines in the intestine of monogastrics (Abreu, 2010). If the results of studies on intestinal tissue can be applied to ruminal tissue, it seems that the increase in expression of cytokines, reported in our previous study (Liu et al., 2013), might be associated with the activation of TLRs during HG diet feeding in goats. Our correlation results also confirmed this inference. However, it is not conclusive whether variations of epimural bacterial community and host TLR genes are the factors that contribute to the initial development of local inflammation in the rumen epithelium during HG diet feeding. Indeed, a very recent study regarding expression of genes and proteins with key protection functions in the rumen epithelium of goats fed low and high grain revealed that a mismatch in gene and protein expression in response to grain level (Hollmann et al., 2013), indicating the upregulation in the expression of TLR genes may not result in an increase in the expression of corresponding proteins. In addition, a previous study showed that a certain degree of upregulation of TLR genes may activate the mucosal innate system in rumen epithelium (Chen et al., 2012), implying the upregulation of TLR gene may be not a negative event for the host and may have a positive effect in preventing damages to the epithelial tissues at some condition. Thus, future studies are needed to determine whether variations in epimural bacterial community are associated with changes in protein expression of host TLRs during HG feeding, as well as how TLRs shape host immune response.

## Conclusion

In general, although a relatively low number of animals were used in the current study, we observed that HG diet feeding causes a strong shift in the epimural bacterial community, and that these changes are associated with alterations in the relative expression of TLRs in the rumen epithelium. This study provides a first insight into the adaptive response of epimural bacterial populations to HG feeding in goats, using the pyrosequencing technique, and shows that these changes were associated with alterations in TLR expression. These findings lead to new insight into host–microbial relationships in ruminants and provide new opportunities to improve ruminant health under the current intensive production system.

## Author contributions

JL carried out the majority of the experiment including animal care, VFA analysis, RNA isolation and real-time PCR; GB, JL and SM were responsible for pyrosequencing data processing, analysis and interpretation; SM and WZ contributed to the conception of the project; The manuscript was prepared by JL and SM.

### Conflict of interest statement

The authors declare that the research was conducted in the absence of any commercial or financial relationships that could be construed as a potential conflict of interest.
