# Supplementary material for: High-grain feeding causes strong shifts in ruminal epithelial bacterial community and expression of Toll-like receptor genes in goats
Source: Front Microbiol. 2015 Mar 2;6:167. doi: 10.3389/fmicb.2015.00167 (PMC4345813; doi:10.3389/fmicb.2015.00167)
Supplement: Supplementary file 1 [file Presentation1.ZIP › 128661_Mao_Supplementary Image_3.PDF]

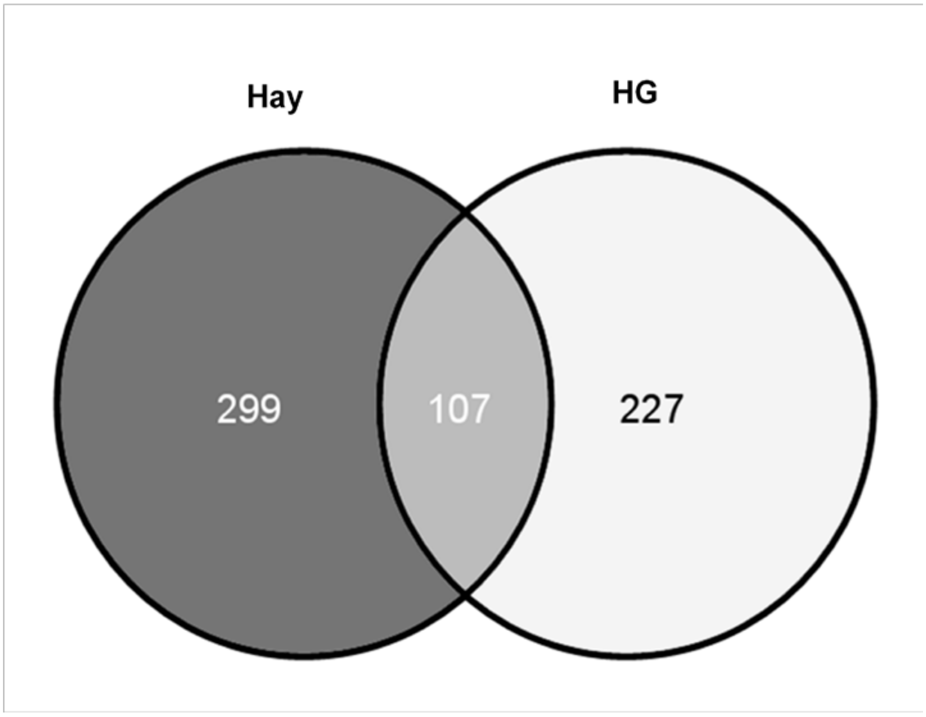

Supplementary figure 3 Shared OTUs across different groups. Venn plot showing the shared and unique OTUs found in each plotted group. Only taxa that are shared by four animals (core) within each group are plotted.
