# Supplementary material for: High-grain feeding causes strong shifts in ruminal epithelial bacterial community and expression of Toll-like receptor genes in goats
Source: Front Microbiol. 2015 Mar 2;6:167. doi: 10.3389/fmicb.2015.00167 (PMC4345813; doi:10.3389/fmicb.2015.00167)
Supplement: Supplementary file 1 [file Presentation1.ZIP › 128661_Mao_Supplementary Image_4.PDF]

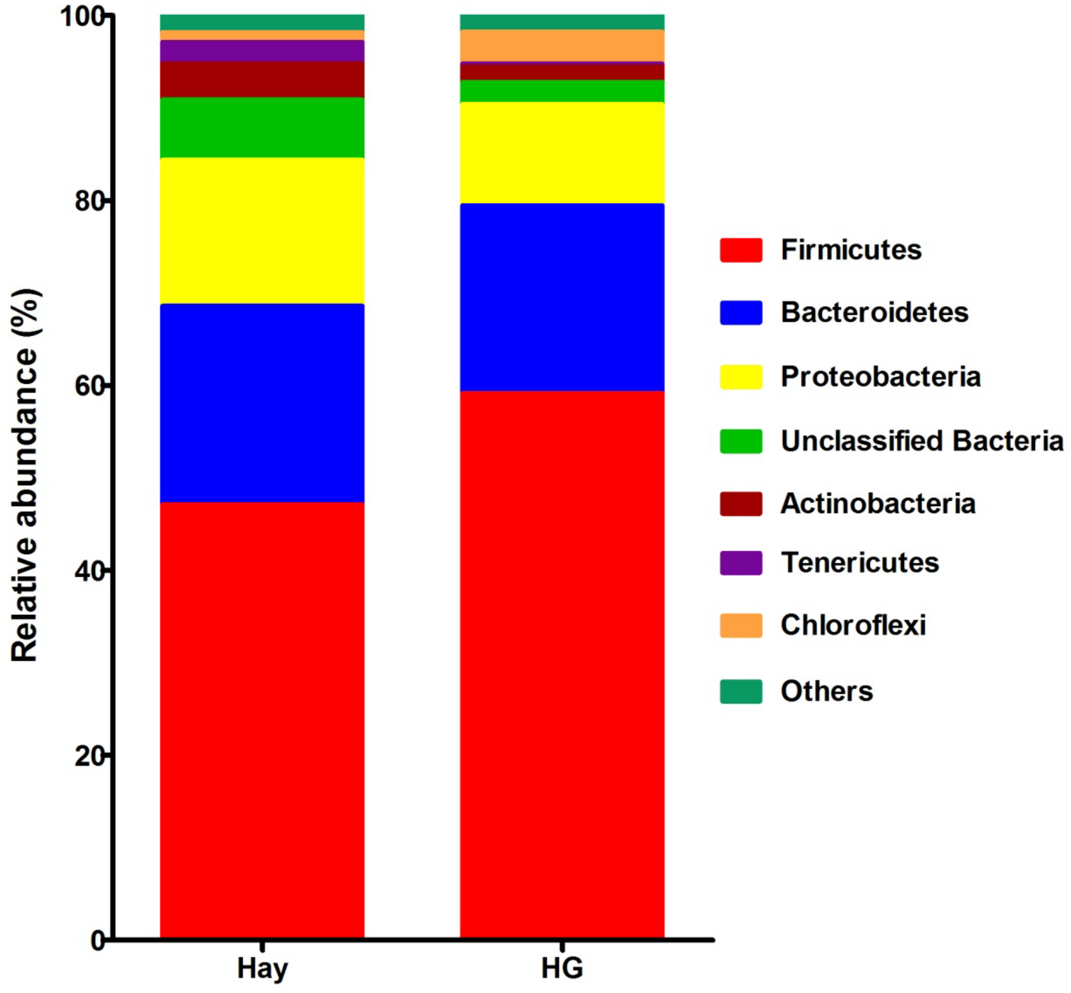

Supplementary figure 4 Percentage contribution of sequences (%) evaluated at the phylum level to the total number of sequences in hay and HG group.
