# Supplementary material for: High-grain feeding causes strong shifts in ruminal epithelial bacterial community and expression of Toll-like receptor genes in goats
Source: Front Microbiol. 2015 Mar 2;6:167. doi: 10.3389/fmicb.2015.00167 (PMC4345813; doi:10.3389/fmicb.2015.00167)
Supplement: Supplementary file 1 [file Presentation1.ZIP › 128661_Mao_Supplementary Image_5.PDF]

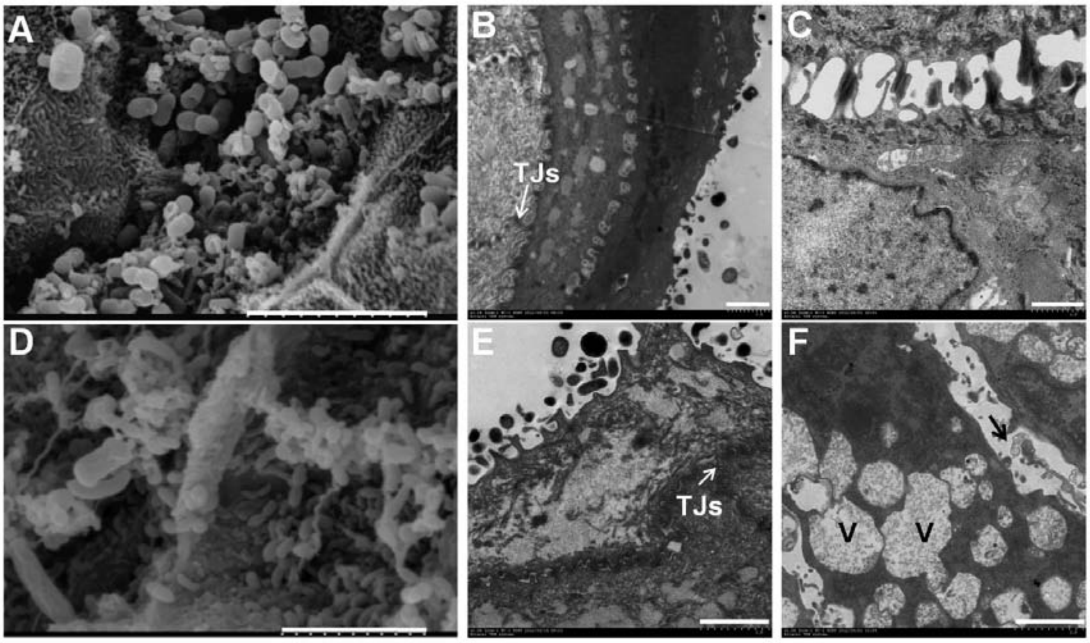

Supplementary figure 5 Rumen papillae morphology and ultrastructure of hay-fed goats (A, B and C) and HG-fed goats (D, E and F). Scanning electron micrograph of rumen papillae surface in hay-fed goats (A; scale bar = 10 μm) and HG-fed goats (D; scale bar = 5 μm). Note the rumen epithelium inhabiting heterogenous microflora in hay and HG group. Transmission electron micrograph of ruminal epithelial corneum and granulosum cell in hay-fed goats (B; scale bar = 1 μm) and HG-fed goats (E; scale bar = 1 μm). Note the strong tight junctions (TJs) in hay-fed goats and diffuse tight junctions in HG-fed goats. Comparisons of ultrastructure of ruminal epithelial granulosum cell in hay-fed goats (C; scale bar = 1 μm) and HG-fed goats (F; scale bar = 2 μm). Note the integrity intercellular junctions in hay-fed goats and declined intercellular junctions and translocation of microbe from rumen into intercellular space (Arrow) in HG-fed goats (V: vacuoles).
