# Supplementary material for: High-grain feeding causes strong shifts in ruminal epithelial bacterial community and expression of Toll-like receptor genes in goats
Source: Front Microbiol. 2015 Mar 2;6:167. doi: 10.3389/fmicb.2015.00167 (PMC4345813; doi:10.3389/fmicb.2015.00167)
Supplement: Supplementary file 1 [file Presentation1.ZIP › 128661_Mao_Supplementary Table_1.DOCX]

**Supplementary table 1** Ingredient and nutrient composition of the experimental diets

| Item | Diet | |
| --- | --- | --- |
|  | Hay | High grain |
| Ingredient composition, % DM | | |
| Chinese *wildrye* | 81.00 | 30.00 |
| Alfalfa | 15.00 | 0 |
| Corn meal | 0 | 45.00 |
| Wheat meal | 0 | 20.00 |
| Soybean | 0 | 1.10 |
| CaCO_3_ | 0.50 | 0.95 |
| NaCl, Salt | 0.80 | 0.65 |
| CaHPO_4_ | 1.70 | 1.20 |
| Mineral and vitamin supplement | 1.00 | 1.00 |
| NaHCO_3_ | 0 | 0.10 |
| Nutrient composition |  | |
| Metabolic energy, MJ/kg DM | 8.31 | 11.31 |
| Crude protein, % DM | 10.06 | 10.06 |
| Crude fat, % DM | 3.55 | 3.59 |
| Crude fiber, % DM | 30.17 | 11.18 |
| Neutral detergent fiber, % DM | 57.01 | 25.23 |
| Acid detergent fiber, % DM | 35.72 | 13.55 |
| Crude ash, % DM | 10.62 | 6.52 |
| Starch, % DM | ND^*^ | 58.23 |

^*^Not determined, but considered equal to 0.
