# Supplementary material for: High-grain feeding causes strong shifts in ruminal epithelial bacterial community and expression of Toll-like receptor genes in goats
Source: Front Microbiol. 2015 Mar 2;6:167. doi: 10.3389/fmicb.2015.00167 (PMC4345813; doi:10.3389/fmicb.2015.00167)
Supplement: Supplementary file 1 [file Presentation1.ZIP › 128661_Mao_Supplementary Table_2.DOCX]

**Supplementary table 2** The effect of high grain (HG) feeding on rumen fermentation at the time of slaughter^a^ (Liu et al., 2013)

| Item | Hay | HG | *P*-value |
| --- | --- | --- | --- |
| ***Ruminal parameters*** |  | | |
| pH | 6.12±0.09 | 5.33±0.09 | <0.001 |
| Total SCFA, mM | 93.90±1.25 | 116.16±4.25 | 0.001 |
| Acetate, mM | 70.24±0.88 | 63.12±1.89 | 0.009 |
| Propionate, mM | 14.57±0.29 | 34.12±2.24 | <0.001 |
| Butyrate, mM | 7.15±0.39 | 15.81±0.94 | <0.001 |
| Isobutyrate, mM | 1.09±0.10 | 1.43±0.15 | 0.089 |
| Valerate, mM | 0.45±0.04 | 0.74±0.05 | 0.002 |
| Isovalerate, mM | 0.41±0.12 | 0.94±0.14 | 0.019 |
| Acetate: Propionate | 4.82±0.05 | 1.88±0.10 | <0.001 |
| Lactic acid, mM | 0.26±0.02 | 0.67±0.04 | <0.001 |
| Free LPS, EU^b^/ml | 22,547±1,065 | 64,628±1,685 | <0.001 |

^a^Values are means ± SE, *n* = 5.

^b^EU= endotoxin unit.

**Reference:**

Liu, J. H., Xu, T. T., Liu, Y. J., Zhu, W. Y. & Mao, S. Y. (2013) A high-grain diet causes massive disruption of ruminal epithelial tight junctions in goats. *Am*. *J*. *Physiol*. *Regul*. *Integr*. *Comp*. *Physiol*. 305, R232-241. doi: 10.1152/ajpregu.00068.2013
