# Supplementary material for: High-grain feeding causes strong shifts in ruminal epithelial bacterial community and expression of Toll-like receptor genes in goats
Source: Front Microbiol. 2015 Mar 2;6:167. doi: 10.3389/fmicb.2015.00167 (PMC4345813; doi:10.3389/fmicb.2015.00167)
Supplement: Supplementary file 1 [file Presentation1.ZIP › 128661_Mao_Supplementary Image_1.PDF]

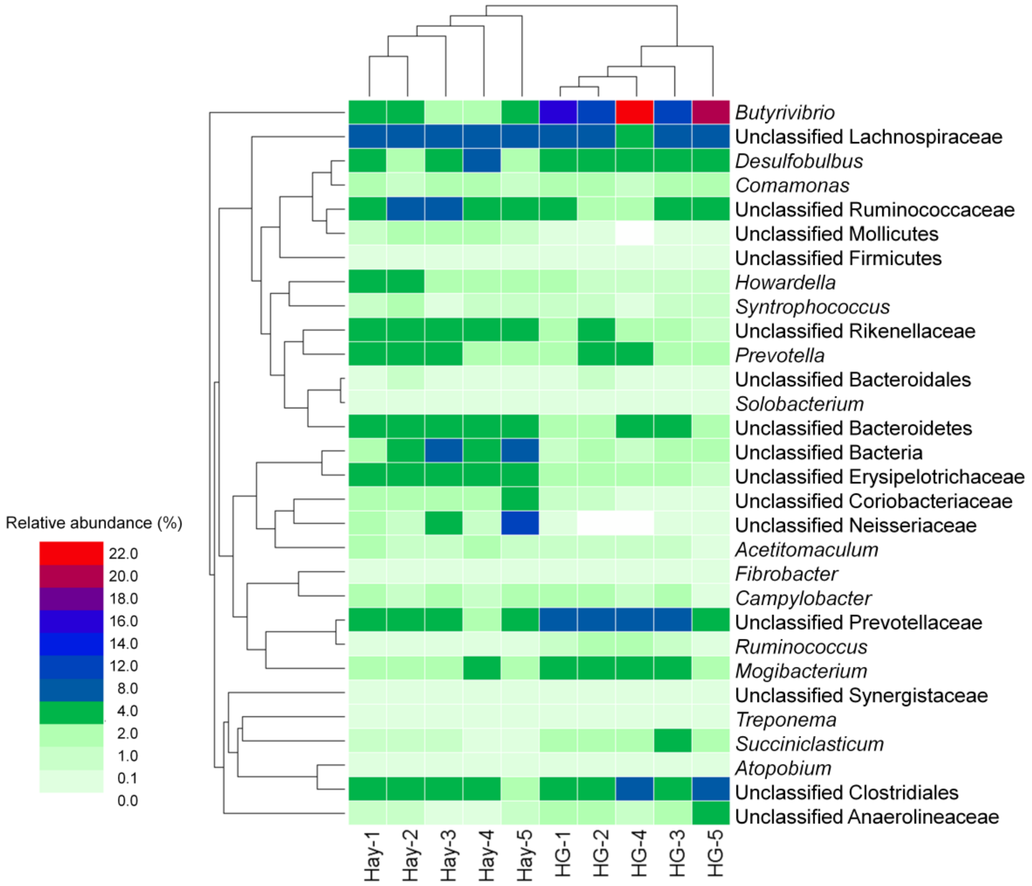

Supplementary figure 1 Heatmap analyses of 30 most abundant genera in hay and HG group. The y axis is a neighbor-joining phylogenetic trees, each row is a different phylotype. The abundance plot shows the proportion of 16S rRNA gene pyrosequences in each sample.
